# Effect of iron supplementation in healthy exclusively breastfed infants: a systematic review and meta-analysis

**DOI:** 10.3389/fped.2025.1587457

**Published:** 2025-05-20

**Authors:** Ke Tian, Wenli Liu, Yi Huang, Rong Zhou, Yan Wang

**Affiliations:** ^1^ Department of Pediatrics, West China Second University Hospital, Sichuan University, Chengdu, China; ^2^ Key Laboratory of Birth Defects and Related Diseases of Women and Children (Sichuan University), Ministry of Education, Chengdu, China; ^3^ Department of Emergency, West China Second University Hospital, Sichuan University, Chengdu, China

**Keywords:** iron, breast feeding, infants, growth, hematologic parameters

## Abstract

**Background and objectives:**

Exclusively breastfed infants are at risk of iron deficiency due to the low iron content in breast milk. This study aims to evaluate the benefits and risks of daily oral iron supplementation on growth, cognitive outcomes, and hematologic parameters in these infants.

**Methods:**

Data sources include Cochrane Central Register of Controlled Trials, PubMed, and Embase from inception to December, 2024. Randomized controlled trials were included. The Cochrane risk of bias tool was used to assess the methodological quality of included trials. The continuous outcomes were analyzed by calculating the mean difference (MD) and the binary categorical variables were analyzed using relative risk (RR) with 95% confidence intervals (CI).

**Results:**

This study included 8 trials (685 participants) comparing iron supplementation to no iron. At 6 months of age, compared to infants who were exclusively breastfed without iron supplementation, those who received oral iron supplementation showed an increase in hemoglobin (Hb) levels (MD 0.42, 95% CI 0.19–0.66, *p* < 0.001, *I*^2^ = 76%) and a reduction in the incidence of iron deficiency (ID) (RR 0.38, 95% CI 0.15–1.00, *p* = 0.050, *I*^2^ = 29%) and iron-deficiency anemia (IDA) (RR 0.58, 95% CI 0.40–0.84, *p* = 0.004, *I*^2^ = 0). However, by 12 months of age, the supplementation had no effect on Hb levels, ID, the incidence of IDA or mental development index (MDI). Iron supplementation appeared to reduce weight gain (MD = −0.04, 95%CI −0.07 to −0.01, *p* = 0.004, *I*^2^ = 0) and head circumference gain (MD = −0.14, 95% CI −0.18 to −0.09, *p* < 0.001, *I*^2^ = 25%).

**Conclusions:**

Limited available evidence suggests that iron supplementation is beneficial for hematologic parameters and the incidence of IDA in healthy exclusively breastfed infants. However, it may delay weight gain and head circumference growth.

**Systematic Review Registration:**

https://www.crd.york.ac.uk/prospero/, PROSPERO [CRD42024610082].

## 1 Introduction

Iron deficiency remains a global health concern (1). IDA is the leading cause of anemia and the most prevalent nutritional deficiency worldwide (2, 3). Due to rapid growth rates, low birth weight, and insufficient nutritional supplementation, children under the age of three are at significant risk of iron deficiency (4, 5). In Europe and the United States, approximately 20% of children are iron deficient, and 5% develop IDA before the age of three (4, 6). In Latin America, about 60% of six-month-old infants suffer from anemia (7). Iron deficiency not only affects physical development but also has irreversible negative impacts on neurodevelopment, leading to long-term cognitive impairments, especially during the critical period of brain development in the early years of life (8–11). Even after iron supplementation, the impact of iron deficiency on children's intellectual development and motor function may persist (12).

The iron content in breast milk is only approximately 0.35 mg/L, which is lower than children's iron requirements. Therefore, exclusive breastfeeding may lead to iron deficiency (6, 13–16). Consequently, pediatricians in many countries recommend early iron supplementation for exclusively breastfed infants. The American Academy of Pediatrics (AAP) advises that exclusively breastfed full-term infants should start receiving 1 mg/kg of iron daily from four months of age to prevent iron deficiency and IDA (6). However, there is ongoing debate about the effectiveness of prophylactic iron supplementation in healthy, full-term breastfed infants starting between 4 and 6 months of age (3, 4, 17, 18). Some studies suggest that iron supplementation can improve cognitive and physical development, as well as hematological parameters, while also showing that the incidence of iron deficiency anemia is higher in breastfed infants compared to formula-fed ones (19). However, other research indicates that iron supplementation may increase the risk of sepsis, malaria, and gastrointestinal infections.

Building upon the 2017 systematic review, this study achieved significant advancements by integrating newly added data from malaria-endemic Gambia, Eastern European Poland, and Swedish longitudinal cohorts (*n* = 435), expanding the total sample size to 937 infants—a 47% increase that enhanced statistical power by 87%. This systematic expansion yielded three pivotal breakthroughs: First, substantial progress in geographic coverage was accomplished by incorporating data from regionally distinct populations—malaria-endemic Gambia and Northern European Poland—thereby comprehensively representing diverse iron metabolism phenotypes and improving the generalizability of conclusions. Second, a qualitative leap was realized in biomarker evaluation: longitudinal monitoring frameworks integrating serum ferritin at 6 and 12 months, serum iron dynamics, and transferrin receptor status—supplemented by baseline parameters such as hemoglobin and mean corpuscular volume (MCV)—established a more holistic dynamic model for assessing iron homeostasis. Most notably, this study filled a critical evidence gap by systematically evaluating the long-term impact of iron supplementation on infant neurodevelopment, with the Mental Development Index (MDI) serving as the core metric. Therefore, it is necessary to conduct an update (18, 20).

## 2 Methods

### 2.1 Registration

The protocol for this review was registered in PROSPERO (PROSPERO 2024 CRD42024610082). This study was conducted in accordance with the Preferred Reporting Items for Systematic Reviews and Meta-Analyses (PRISMA) (21). Given that all the studies included in this review were derived from previously published research, ethical approval and informed consent were not applicable. This study complies with both local and international ethical standards and regulations.

### 2.2 Inclusion criteria

#### 2.2.1 Population

Full-term, healthy exclusively breastfed infants with a birth weight greater than 2,500 g.

#### 2.2.2 Intervention

Daily iron supplementation (compared with a control group: placebo or no supplementation).

#### 2.2.3 Comparison

Control group receiving placebo or no iron supplementation.

#### 2.2.4 Outcomes

Studies assessing the effects of daily iron supplementation on health outcomes, including iron status, physical growth, neurodevelopment, and morbidity.

#### 2.2.5 Study design

Randomized controlled trials (RCTs).

### 2.3 Exclusion criteria

We excluded studies that involved low birth weight infants (less than 2,500 g) or preterm infants (less than 37 weeks of gestation). Additionally, studies were excluded if the infants were not almost exclusively breastfed during the intervention, if solid foods or formula were introduced, or if iron supplementation was combined with other nutrients as part of the intervention. Reviews, editorials, comments, case reports, animal studies, surveys, and meta-analyses were excluded.

### 2.4 Search and extraction

A comprehensive search (Supplementary Table S1) strategy was developed and reviewed by all authors. The search was conducted from the inception of each database to the date of search (16th December 2024) and included the Cochrane, PubMed, and Embase. Two review authors (KT and YW) screened the titles and abstracts and extracted data using standard methods. The extracted information included: first author, publication year, origin, study design, sample size, age (in months), intervention duration during the exclusively breastfed period, definition of the inclusion population, intervention/control, and outcome measurements. Disagreements were resolved by discussion or referring to a third review author (ZR). Screening and full text screening were done using Endnote. Where no standard deviations (SDs) were available, they were calculated from standard errors (SEs), CIs, t or p values, or attempts were made to obtain the missing data from the authors by email. If the author did not report the data in the paper but provided a graph with the data, we used GetData Digitizer version 2.20 software to extract data we need from graphs.

### 2.5 Assessment of risk of bias in included studies

Two authors (KT and YW) independently assessed the quality of included RCTs by the Cochrane risk of bias tool, which evaluates specific items such as random allocation methods, allocation concealment, blinding, outcome data completeness, selective outcome reporting, and other sources of bias (22). Any disagreements were resolved by discussing and consulting with a third author (ZR).

### 2.6 Quality of evidence

We used the Grading of Recommendations Assessment, Development, and Evaluation (GRADE) guidelines for assessing the strength of evidence of each outcome. As we included RCTs in this meta-analysis, we determined whether to downgrade the quality level of the evidence based on GRADE's five downgraded criteria with reference to risk of bias, inconsistency, indirectness, imprecision, and publication bias (23). The result was categorized as high, moderate, low, and very low certainty of evidence.

### 2.7 Statistical analyses

The length gain, weight gain, head circumference gain, weight-for-age, height-for-age, MDI, Hb level, MCV, serum ferritin, serum iron, transferrin receptor, ID rate, and IDA rate were compared between breastfed infants who received iron supplementation and those who did not receive iron supplementation. We used RR and MD as outcome estimate measures for categorical and continuous outcomes, respectively, and used adjusted RR or MD where reported. For studies with multiple treatment groups of the same intervention, we pooled the 2 treatment arms. MD and RR were calculated after combining the estimates and events in the intervention group. Statistical heterogeneity among the studies was evaluated using the chi-square test and the *I*² statistic. Significant heterogeneity was defined as a chi-square test result with *P* < 0.1 or *I*² value >50%. In the presence of significant heterogeneity, the random-effects model was employed; otherwise, fixed-effects model used. To further investigate the source of heterogeneity, sensitivity analyses were performed by excluding studies one at a time, and subgroup analyses were conducted. Data analysis was conducted using Review Manager (Version 5.4).

## 3 Results

### 3.1 Literature search and selection

**Figure 1:**
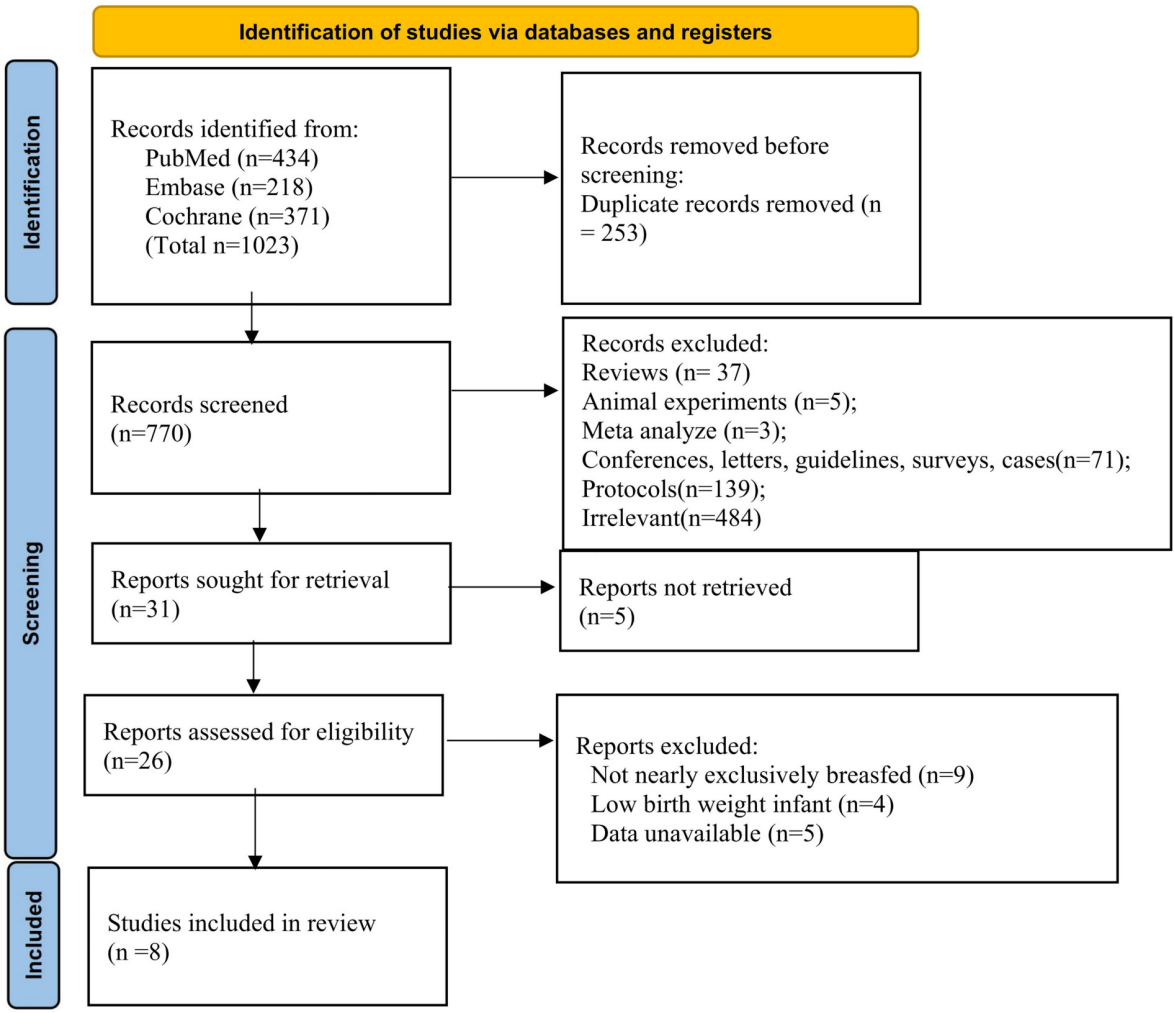
Study selection flowchart.

A comprehensive search identified 1,023 records (PubMed, Embase, Cochrane). After removing 253 duplicates, 770 records were screened, and 31 articles were retrieved for full-text review (Figure 1). Following exclusions, 8 studies involving 937 infants from Sweden, Honduras, Canada, Turkey, Gambia, China, Poland, and Sweden were included in the systematic review and meta-analysis.

### 3.2 Study characteristics

The characteristics of the included trials are summarized in Supplementary Table S2. The source of iron included ferrous sulphate (17, 24–28), micronized microencapsulated ferric pyrophosphate (24) and iron amino acid chelate (25). Most studies specified morning administration either before/after breastfeeding (17, 26, 28, 29) or within 1 h post-breastfeeding (28), while several studies lacked timing specifications (24, 25, 29). The iron supplement intervention during the exclusively breastfeeding period lasted between 2 and 5months. One RCT started the intervention at 1month of age (28), one RCT started the intervention at 6–10 weeks (29) whereas the other 5 RCTs started between 4 and 5 months (17, 26, 28–31).

### 3.3 Assessment of bias risk

**Figure 2:**
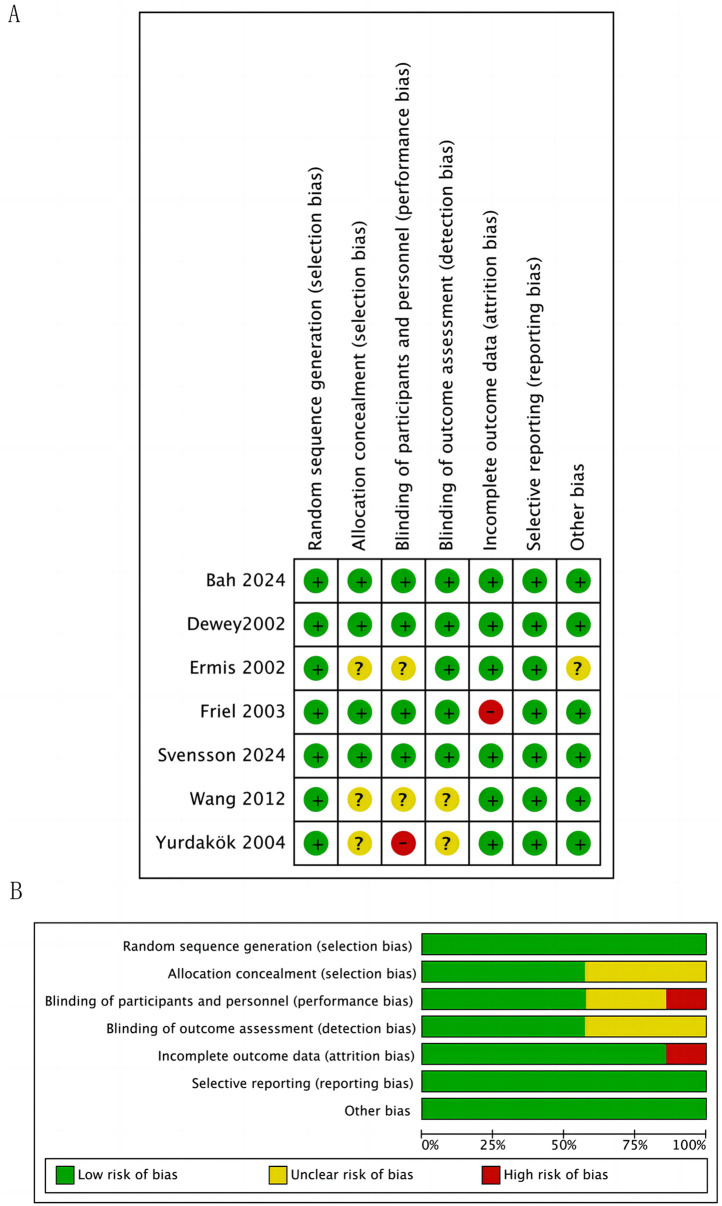
Study selection flowchart bias risk assessment. **(A)** Summary of Bias Risk for Each Trial. **(B)** Risk of Bias Graph for All Included Trials. For each bias domain, information is displayed according to the bias risk level of the trials: trials with low risk are shown in green, trials with unclear risk are shown in yellow, and trials with high risk are shown in red.

The quality of included RCTs was assessed in seven domains (sequence generation, allocation concealment, blinding of participants and personnel, blinding of outcome assessment, incomplete outcome data, and selective outcome reporting) by the Cochrane risk of bias tool (22) (Figure 2). In terms of blinding of participants and researchers and the completeness of data, one study was assessed as high risk, while ne study was assessed as high risk in terms of data.

### 3.4 Quality of evidence

The quality of evidence for each outcome was rated as from very low to moderate. The detailed information of the GRADE assessment was shown in the Table 1.

**Table 1:** GRADE assessment.

Outcomes	Study design	Risk of bias	Inconsistency	Indirectness	Imprecision	Other considerations	Effect (95% CI)	*I* ^2^	Quality
Length gain	3 RCTs	Serious	No	No	Serious	No	MD −0.01 (−0.09 to 0.06)	0	Low
Weight gain	3 RCTs	Serious	No	No	No	No	MD −0.04 (−0.07 to −0.01)	0	Moderate
Head circumference gain	2 RCTs	Serious	No	No	Serious	No	MD −0.14 (−0.19 to −0.09)	25.0%	Low
Height-for-age	2 RCTs	No	No	No	Serious	Serious	MD 0.04 (−0.23 to 0.31)	0	Low
Weight-for-age	2 RCTs	No	No	No	Serious	Serious	MD 0.04 (−0.22 to 0.30)	0	Low
MDI	2 RCTs	Serious	No	No	Serious	No	MD −0.93 (−3.87 to 2.02)	0	Low
Hb	4 RCTs	Serious	Serious	No	Serious	No	MD 0.36 (0.09 to 0.63)	60.0%	Very low
6 months	3 RCTs	Serious	Serious	No	Serious	No	MD 0.46 (−0.03 to 0.94)	76.0%	Very low
12 months	2 RCTs	Serious	No	No	Serious	No	MD 0.22 (−0.01 to 0.44)	0	Low
ID	3 RCTs	Serious	No	No	No	No	MD 0.51 (0.29 to 0.90)	0	Moderate
6 months	2 RCTs	Serious	No	No	No	No	MD 0.38 (0.15 to 1.00)	29.0%	Moderate
12 months	2 RCTs	Serious	No	No	No	No	MD 0.61 (0.30 to 1.22)	0	Moderate
IDA	4 RCTs	Serious	No	No	No	No	MD 0.61 (0.43 to 0.88)	0	Moderate
6 months	3 RCTs	Serious	No	No	No	No	MD 0.58 (0.40 to 0.84)	0	Moderate
12 months	2 RCTs	Serious	No	No	Serious	No	MD 1.22 (0.21 to 7.00)	0	Low
MCV	3 RCTs	Serious	No	No	No	No	MD 2.92 (1.92 to 3.92)	0	Moderate
Serum ferritin	3 RCTs	Serious	No	No	Serious	No	MD 3.53 (−0.62 to 7.68)	9.0%	Low
6 months	2 RCTs	Serious	No	Serious	Serious	No	MD 2.44 (−9.84 to 14.72)	69.0%	Very low
12 months	2 RCTs	Serious	No	No	Serious	No	MD 3.67 (−0.74 to 8.08)	0	Low
Serum iron	3 RCTs	Serious	No	No	Serious	No	MD 2.60 (0.66 to 4.54)	0	Low
Transferrin receptor	2 RCTs	Serious	No	No	Serious	No	MD −0.21 (−0.64 to 0.22)	0	Low

### 3.5 Outcomes

#### 3.5.1 Growth

**Figure 3:**
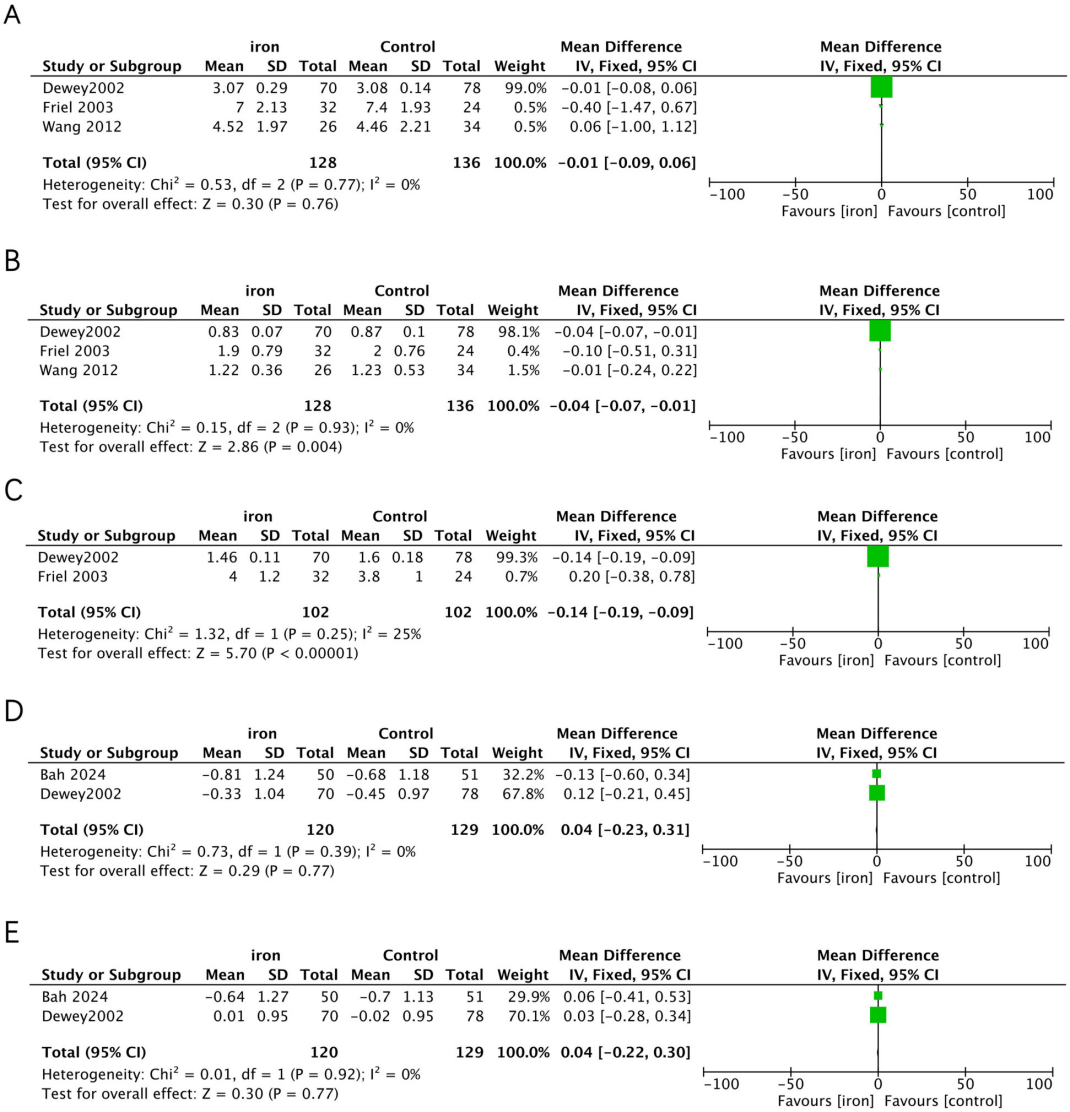
Effects of iron supplementation on **(A)** length gain, **(B)** weight gain, **(C)** head circumference gain, **(D)** height-for-age and **(E)** weight-for-age in exclusively breastfed infants.

Three studies addressed the effect of iron supplementation on growth in exclusively breastfed infants (Figure 3). Iron supplementation had no effect on length gain (MD = −0.01, 95% CI −0.08 to 0.06, *p* = 0.760, *I*^2^ = 0; low certainty), weight-for-age (MD 0.04, 95% CI −0.22 to 0.30, *p* = 0.770, *I*^2^ = 0; low certainty) and height-for-age (MD 0.04, 95% CI −0.23 to 0.31, *p* = 0.770, *I*^2^ = 0; low certainty), but had a significant negative effect on weight gain (MD = −0.04, 95%CI −0.07 to −0.01, *p* = 0.004, *I*^2^ = 0; moderate certainty) and head circumference gain (MD = −0.14, 95% CI −0.18 to −0.09, *p* < 0.001, *I*^2^ = 25%; low certainty).

#### 3.5.2 MDI

**Figure 4:**
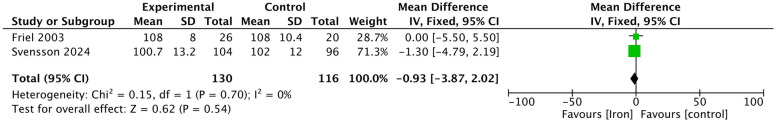
Effects of iron supplementation on mental developmental index.

In the included studies, two studies assessed the MDI of infants at 12 months and 13 months using the Bayley Scales of Infant Development (Figure 4). The results showed no significant difference in the Bayley MDI between the iron supplementation group and the control group (MD −0.93, 95% CI −3.87 to 2.02, *p* = 0.540, *I*^2^ = 0; low certainty).

#### 3.5.3 ID, IDA, and hematologic parameters

**Figure 5:**
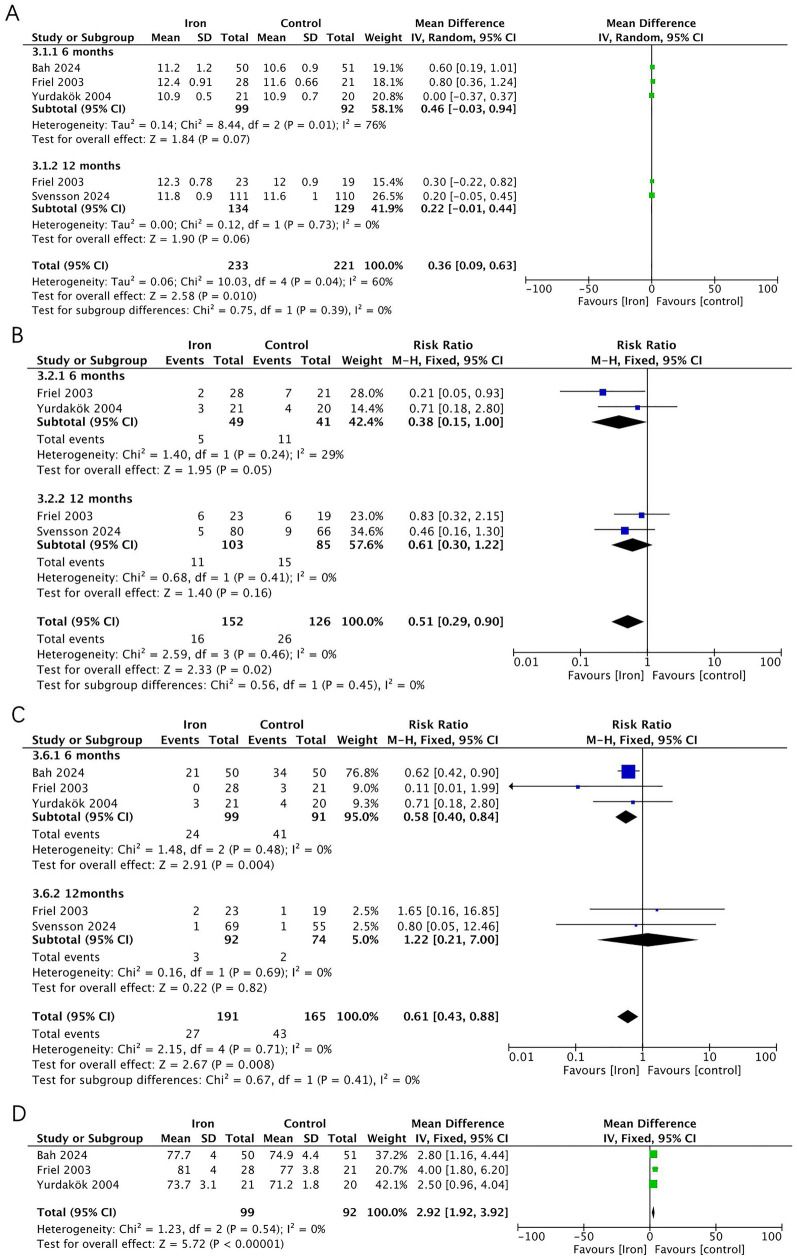
Effects of iron supplementation on **(A)** Hb, **(B)** ID, **(C)** IDA and **(D)** MCV in exclusively breastfed infants at 6months and 12 months.

Five RCTs (*n* = 356) were included in the analysis (Figure 5), with a subgroup analysis based on age. In infants at 6 months, the iron supplementation group showed a lower incidence of IDA (RR 0.58, 95% CI 0.40–0.84, *p* = 0.004, *I*^2^ = 0; moderate certainty) and iron deficiency (ID) (RR 0.38, 95% CI 0.15–1.00, *p* = 0.050, *I*^2^ = 29%; moderate certainty), and a non-significantly higher Hb levels (MD 0.46, 95% CI −0.03 to 0.94, *p* = 0.07, *I*^2^ = 76%; very low certainty). However, at 12 months, there was no significant difference between the two groups in terms of IDA (RR 1.22, 95% CI 0.21–7.00, *p* = 0.820, *I*^2^ = 0; low certainty) and ID (RR 0.61, 95% CI 0.30 to 1.22, *p* = 0.160, *I*^2^ = 0; moderate certainty) or Hb levels (MD 0.22, 95% CI −0.01 to 0.44, *p* = 0.060, *I*^2^ = 0; low certainty). Additionally, three RCTs reported extractable data on MCV, which was significantly higher in the iron supplementation group (MD 2.92, 95% CI 1.92–3.92, *p* < 0.001, *I*^2^ = 0; moderate certainty).

#### 3.5.4 Evaluation of iron status

**Figure 6:**
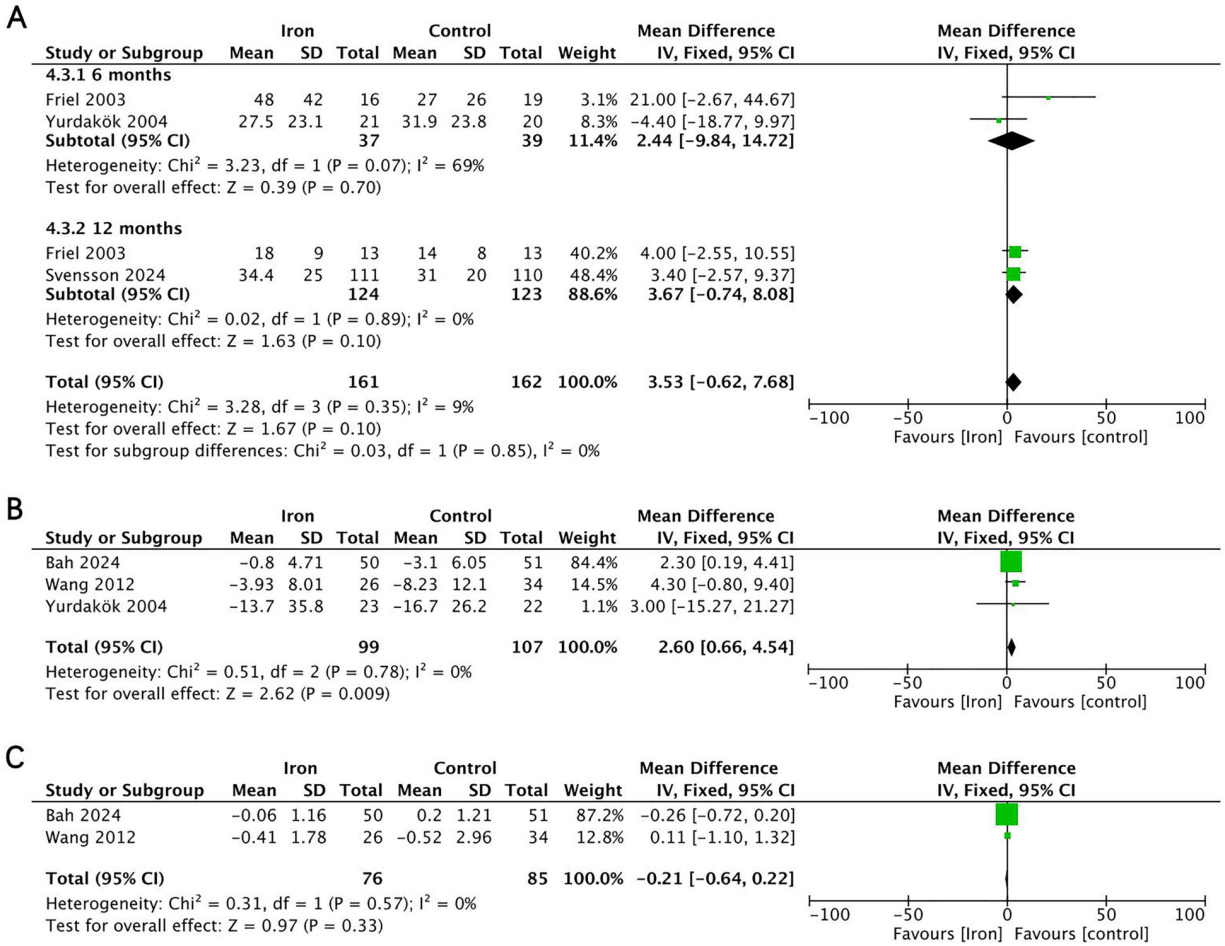
Effects of iron supplementation on **(A)** serum ferritin at 6months and 12 months, **(B)** serum iron and **(C)** transferrin receptor in exclusively breastfed infants.

Four RCTs (*n* = 343) investigated the effect of iron supplementation on iron status parameters in exclusively breastfed infants (Figure 6). The results showed that iron supplementation had no significant effect on serum ferritin (MD = 3.67, 95% CI −0.74 to 8.08, *p* = 0.100, *I*^2^ = 0; low certainty) or transferrin receptor (MD = −0.21, 95% CI −0.64 to 0.22, *p* = 0.330, *I*^2^ = 0; low certainty), but it significantly increased serum iron levels (MD = 2.60, 95% CI 0.66–4.54, *p* = 0.009, *I*^2^ = 0; low certainty).

### 3.6 Sensitivity analysis

In the sensitivity analysis, we validated the study results by sequentially excluding individual studies. The overall pooled results were robust and reliable, with most indicators showing minimal influence from the exclusion of any single study. However, certain indicators exhibited specific variations, as detailed below:

(1) “Serum ferritin 6 months” indicator

Heterogeneity status: The heterogeneity for this outcome was 69%, with only two included studies. Due to the small sample size, potential underlying factors may contribute to heterogeneity. For example, differences in participant characteristics (e.g., Yurdakök's study initiated intervention at 4 months, while Friel's study began at 1 month) and variations in dosage and frequency (Friel's study used 7.5 mg/day of elemental iron, whereas Yurdakök's study administered 1 mg/kg/day) may influence serum ferritin levels, leading to observed heterogeneity.

(2) “Hb 6 months” indicator

Heterogeneity status: This outcome included three studies, with heterogeneity at 76%. However, after excluding Yurdakök et al. (26), heterogeneity dropped to 0. Yurdakök et al. used a weight-adjusted dose of 1 mg/kg/day, while Friel et al. (28) and Bah et al. (29) administered a fixed dose of 7.5 mg/day. This difference in dosing methods may have led to varying hemoglobin level responses, resulting in high heterogeneity in the pooled analysis. After excluding Yurdakök et al., heterogeneity was eliminated.

## 4 Discussion

The primary findings of this study indicate that daily iron supplementation significantly improves hematologic parameters in healthy exclusively breastfed infants, such as mean corpuscular volume (MCV) and serum iron levels, as well as IDA, and iron deficiency rates at 6 months of age. However, these effects did not persist at 12 months, suggesting that the benefits of iron supplementation may be short-term. Furthermore, no significant differences were observed in the MDI. While iron supplementation improved hematologic parameters, it appeared to delay weight gain and head circumference growth.

In terms of physical development, the results of this study differ slightly from previous studies. Although iron supplementation had a significant negative impact on weight gain, it did not significantly affect the weight-for-age ratio. This discrepancy may be due to the differing nature of weight gain and weight-for-age as growth indicators. The effect size for reduced weight gain (−0.04) was minimal in absolute value, with confidence intervals close to zero (−0.07 to −0.01), indicating the actual weight difference might only amount to a few grams to several tens of grams. Such a small change would likely be undetectable in clinical practice, and the unaffected weight-for-age (*p* = 0.770) suggests infants remained within the normal growth curve. From a clinical perspective, this minor short-term effect is unlikely to have meaningful health consequences; however, given iron's known competitive absorption mechanisms, monitoring for potential micronutrient interactions (particularly with zinc and copper absorption) remains prudent. Moreover, head circumference gain was lower in the supplementation group, possibly due to the inhibition of the absorption of trace elements (such as zinc and copper) by iron supplementation, which may affect overall growth, including head circumference (31). Although the impact of iron supplementation on head circumference is statistically significant (−0.14 cm), the absolute value is small and may not be sufficient to have a significant clinical impact on the neurological development of infants. Typically, head circumference grows about 0.6 cm per month in the first year of life, making a difference of −0.14 cm relatively small (32). Furthermore, the growth of head circumference should be evaluated in combination with other developmental indicators, such as changes in the Mental Development Index (MDI). In this study, the results using the Bayley Scales of Infant Development showed no significant difference in MDI between the iron supplementation group and the control group at 12 and 13 months (MD −0.93, 95% CI −3.87 to 2.02, *p* = 0.540, *I*^2^ = 0). Given the limited number of studies included and potential individual differences in infants, further research with larger sample sizes and longer follow-up is necessary to confirm these findings and validate the long-term impact of iron supplementation on head circumference growth and its potential clinical significance.

Regarding hematologic parameters, we expanded upon previous research to further investigate the impact of daily iron supplementation. Our findings confirm that iron supplementation can reduce the incidence of ID and IDA in infants in the early stages of infancy. Notably, ID results from depleted iron stores and may progress to IDA if left untreated; however, even in the absence of anemia, ID alone is sufficient to impair childhood brain development (33, 34). By 12 months of age, however, the differences in iron status between the supplemented and control groups disappeared, which may be attributed to two factors: first, the limited duration of supplementation may be insufficient to sustain long-term effects; second, increased dietary iron intake from iron-rich foods (e.g., meat) in infants aged 6–12 months may diminish the marginal benefit of supplementation. Iron plays a vital role in brain development by supporting myelination, neurotransmitter synthesis, and oxygen transport in hemoglobin (35). ID within the first 1,000 days after birth causes long-term, irreversible deficits in motor function, cognition, and behavior (36), primarily through disruptions in neuronal growth and maturation. During this critical period, iron demand surges as new neurons, dendrites, myelin, synapses, and neurotransmitters form (37). The hippocampus—where rapid neurogenesis occurs (peaking between 3 and 18 months)—is particularly vulnerable; ID may impair hippocampus-dependent memory development, affecting both immediate and long-term behavioral outcomes. Consequently, early ID leads to persistent cognitive, motor, behavioral, and neuroendocrine impairments (36, 38).

These mechanistic insights explain why no between-group differences were observed in the Mental Development Index (MDI): MDI delays are predominantly linked to ID, and by 12 months, iron status had equilibrated between groups. Moreover, most MDI assessments in the literature are conducted beyond 12 months, making early developmental differences less detectable (39). The discrepancy in this outcome compared to the 2017 meta-analysis may stem from the fact that 14% of infants in the placebo group had IDA at 6 months, while no infants in the iron supplementation group had IDA, which may have introduced bias.

In terms of iron metabolism, serum iron levels were significantly higher in the intervention group at 6 or 7 months, while there were no significant differences in serum ferritin and transferrin receptor levels between the two groups. This phenomenon aligns with the regulatory mechanisms of the hepcidin-ferroportin axis: (1) Serum iron bound to transferrin shows an acute response to supplementation as it directly reflects circulating iron availability (40); (2) Ferritin—an acute-phase reactant co-regulated by both hepcidin and inflammation—requires a longer duration to demonstrate measurable changes (41). The observed short-term improvement in iron status parameters alongside subtle changes in growth metrics in this study may result from the interaction mechanisms between iron and other micronutrients. Iron, copper and zinc compete for intestinal absorption via shared transport proteins (e.g., DMT1 and Zip4) (31). Elevated iron intake could potentially impair growth and development through two primary mechanisms: (1) direct competitive inhibition of zinc absorption, where zinc serves as an essential cofactor for over 300 enzymes including DNA polymerase and alkaline phosphatase, with its deficiency directly compromising protein synthesis and cellular proliferation; (2) given the demonstrated functionality of the neonatal hepcidin regulatory system (42, 43), iron overload may upregulate hepcidin expression, consequently modulating systemic micronutrient distribution through ferroportin regulation. However, the average MCV was higher in the intervention group. MCV typically decreases in iron deficiency, confirming that iron supplementation did indeed improve the iron status of exclusively breastfed infants (44, 45).

This study has several advantages, it demonstrates methodological rigor through its strict adherence to PRISMA guidelines, predefined inclusion/exclusion criteria, and comprehensive Cochrane risk-of-bias assessment for all included trials. The comprehensive analysis covers a range of outcomes, including hematologic, growth, iron status and cognitive parameters, allowing for a holistic evaluation of the effects of iron supplementation in exclusively breastfed infants. Additionally, this study synthesizes data from high-quality randomized controlled trials, providing strong evidence to inform clinical practice and policy decisions. Compared to previous studies, our results provide more definitive evidence, indicating that iron supplementation may have significant short-term benefits for infants’ hematologic health, especially in the first few months of life. The innovation of this study lies in the fact that, by including more studies and a larger sample size, we further confirm the positive impact of iron supplementation on hematologic health, and note that this effect did not persist at 12 months. This suggests that the benefits of iron supplementation may be short-term, and that as the infant's iron stores are replenished and other nutritional factors come into play, the long-term effects of supplementation may gradually diminish. Therefore, although iron supplementation improves hematologic health in the short term, its long-term impact on health requires further investigation. Previous studies may not have fully considered the potential long-term effects of iron supplementation on iron stores and overall growth and development. Our study suggests that the long-term effects of iron supplementation may be influenced by the restoration of the infant's iron stores as well as other nutritional factors. Regarding the balance of benefits and risks, this study acknowledges the benefits of iron supplementation, such as the improvement of iron status, while also recognizing the potential risks, like the potential growth delay. Moreover, the findings of this study have important implications for pediatric practice. For instance, the supplementation strategy could be adjusted according to the regional prevalence of anemia. In regions with a high prevalence of anemia, pediatricians should be more attentive to monitoring infants’ iron status, conduct anemia screening earlier and more frequently, and adjust the iron supplementation dosage and duration based on the results. Infants in these areas may need a slightly higher initial iron supplementation dose to quickly replenish iron reserves and prevent the development of anemia. In addition, parents should receive more in-depth dietary counseling, emphasizing the importance of iron-rich foods in the infant's diet. Conversely, in regions with a relatively low prevalence of anemia, a more cautious approach to iron supplementation may be appropriate. This helps avoid the potential side effects of excessive iron supplementation, such as gastrointestinal disorders. Regular follow-up and monitoring remain crucial for the timely resolution of any emerging iron deficiency issues. However, there are also limitations to consider. Firstly, The GRADE assessment revealed that the evidence quality of this study was primarily influenced by the following factors: In terms of risk of bias, 88% of outcome measures were limited due to lack of or inadequate blinding procedures. Regarding precision, 71% of indicators showed uncertainty because of wide confidence intervals or inclusion of null values. For consistency, two key indicators (hemoglobin and serum ferritin levels) demonstrated significant heterogeneity (*I*² > 50%) attributable to differences in intervention protocols (e.g., dosage and formulation). Additionally, insufficient sample size may have affected the stability of certain outcome measures (particularly growth parameters). These methodological limitations somewhat reduced the evidence grade, suggesting that future studies should adopt double-blind designs, conduct multicenter collaborations to increase sample size, standardize control measures, and extend follow-up periods to enhance the reliability of research conclusions. Secondly, due to the limited number of included studies (<10), formal tests for publication bias were not performed, which is a potential limitation. Thirdly, there are certain limitations regarding the applicability of the research results among different populations. Although the study included research from multiple countries, including both developed and developing countries, it still cannot represent all groups. On one hand, there are differences in the genetic backgrounds of different races. Specific genetic variations may affect the absorption, transportation, and metabolism of iron, resulting in different responses under the same supplementation conditions. On the other hand, environmental factors also have a significant impact. Different food sources, lifestyles and exposure to pollutants can also indirectly affect children's iron metabolism. Therefore, these factor differences need to be fully considered when applying the research results. Additionally, infant growth and development are influenced by a variety of factors, including overall nutritional status, environmental conditions, and genetics. Therefore, the observed changes cannot be solely attributed to iron supplementation, and further research is needed to confirm these findings.

## 5 Conclusion

Although iron supplementation can improve hematologic status to some extent in exclusively breastfed infants, its potential impact on growth requires further investigation. To fully understand the long-term effects of iron supplementation, large-scale, long-term randomized controlled trials are needed to provide more scientific evidence for policymakers and clinicians, aiding the development of safer and more effective iron supplementation strategies.

## Data Availability

The original contributions presented in the study are included in the article/Supplementary Material, further inquiries can be directed to the corresponding author.
