# Supplementary material for: Effect of iron supplementation in healthy exclusively breastfed infants: a systematic review and meta-analysis
Source: Front Pediatr. 2025 May 20;13:1587457. doi: 10.3389/fped.2025.1587457 (PMC12129980; doi:10.3389/fped.2025.1587457)
Supplement: Supplementary file 1 [file Table1.docx]

S1 table. Search strategy for Pubmed

| Sequence | Query |
| --- | --- |
| #1 | Breast Feeding (Mesh) |
| #2 | Breastfeeding |
| #3 | Breastfed |
| #4 | Breast Feeding, Exclusive |
| #5 | Exclusive Breast Feeding |
| #6 | Breastfeeding, Exclusive |
| #7 | Exclusive Breastfeeding |
| #8 | Wet Nursing |
| #9 | Milk Sharing |
| #10 | Sharing, Milk |
| #11 | Wet-Nursing |
| #12 | WetNursing |
| #13 | MilkSharing |
| #14 | BreastfeedingExclusive |
| #15 | ExclusiveBreastfeeding |
| #16 | #1 or #2 or #3 or #4 or #5 or #6 or #7 or #8 or #9 or #10 or #11 or #12 or #13 or #14 or #15 |
| #17 | Iron (Mesh) |
| #18 | Iron 56 |
| #19 | Iron-56 |
| #20 | #17 or #18 or #19 |
| #21 | Clinical trials (Mesh) |
| #22 | Randomized Clinical trials |
| #23 | Randomized |
| #24 | Random |
| #25 | Placebo |
| #26 | Trial |
| #27 | Groups |
| #28 | #21 or #22 or #23 or #24 or #25 or #26 or #27 |
| #29 | #16 AND #20 AND #28 |
