# Supplementary material for: Effect of iron supplementation in healthy exclusively breastfed infants: a systematic review and meta-analysis
Source: Front Pediatr. 2025 May 20;13:1587457. doi: 10.3389/fped.2025.1587457 (PMC12129980; doi:10.3389/fped.2025.1587457)
Supplement: Supplementary file 2 [file Table2.pdf]

S2 table. Summary of Studies Included in the Meta-Analysis

Study	Origin	Study Design	Sample Size	Age (month)	Intervention Duration during the exclusively breastfed period	Definition of Inclusion Population	Intervention details	Control details	Outcome Measurements
Dewey,2002	Sweden	RCT	101	4	2	exclusively or nearly exclusively breastfed to 6 months	1 mg/kg/d ferrous sulfate; single AM dose post-breastfeeding	Unkown； identical timing protocol	Growth Iron status
Domellof,2001	Honduras		131						
Ermis,2002	Turkey	RCT	113	5	4	exclusively breastfed to 9 months	1 mg/kg/day ferrous sulfate；single AM dose pre/post-breastfeeding	placebo；identical timing protocol	Iron status
Friel,2003	Canada	RCT	77	1	5	exclusively breastfed to 6 months	7.5 mg/d ferrous sulfate；Once daily within 1h of breastfeeding	iron-free drops; identical timing protocol	Growth Iron status Mental development
Yurdakök,2004	Turkey	RCT	70	4	3	exclusively breastfed to 7months	1 mg/kg/d ferrous sulfate；single AM dose pre/post-breastfeeding	iron-free drops; identical timing	Growth Iron status

								protocol	
Wang,2012	China	RCT	123	4	2	exclusively breastfed to 6 months	1 mg/kg of iron supplementation (amino acid chelated iron) daily	No iron；identical timing protocol	Growth<br>Iron status
Bah,2024	Gambia	RCT	101	6-10 weeks	14weeks	exclusively breastfed to 20-24 months	7.5 mg / day of iron in 0.5 mL of 70% sorbitol solution;<br>once daily without specification of timing	0.5 mL of 70% sorbitol solution；identical protocol	Iron status
Svensson，2024	Poland and Sweden	RCT	221	4	5	exclusively or nearly exclusively breastfed to 9 months	1 mg/kg/d of iron supplementation(micronized microencapsulated ferric pyrophosphate)；<br>once daily without specification of timing	Maltodextrin；identical protocol	Iron status<br>Mental development
